# Microvesicles derived from bone marrow and peripheral blood can reflect tumor load in multiple myeloma

**DOI:** 10.1515/jtim-2025-0096

**Published:** 2025-12-29

**Authors:** Nanhao Meng, Zhaoyun Liu, Yan Shi, Chun Yang, Hao Wang, Yanran Luo, Hui Liu, Kai Ding, Fengjuan Jiang, Fengping Peng, Rong Fu

**Affiliations:** Department of Hematology, Tianjin Medical University General Hospital, Tianjin, China; Tianjin Key Laboratory of Bone Marrow Failure and Malignant Hemopoietic Clone Control, Tianjin Institute of Hematology, State Key Laboratory of Experimental Hematology, Tianjin, China

## To the editor

Multiple myeloma (MM) is a malignant tumor of plasma cells that cannot be completely cured. Although the development of revolutionary immunotherapies has dramatically improved outcomes of MM patients,^[1]^ the residual tumor load often leads to relapse in patients who have achieved complete response (CR).^[2]^ Currently, the multicolor flow cytometry and next-generation sequencing are mainly used to monitor MM tumor load in clinical practice, in particular, the EuroFlow consortium has developed a highly sensitive and fully standardized approach using the next-generation flow (NGF).^[3]^ However, due to the focal distribution of myeloma cells in the bone marrow cavity, these existing methods may show false-negative test results.^[3]^ To address this possible false negative situation, we tried to use circulating microvesicles to reflect MM tumor load. In previous studies, we have found that the microvesicles derived from MM cells in the bone marrow can reflect MM tumor load.^[4]^ Furthermore, this was the first study to explore paired bone marrow and peripheral blood samples and the first one to investigate the value of GPRC5D and BCMA double-positive microvesicles in monitoring MM tumor load.

This study included 311 MM patients (Supplementary Table S1) with tumor load assessed by NGF, and whose clinical efficacies were evaluated as CR. Patients were grouped into a high tumor load (H-TL) and a low tumor load (L-TL) group based on NGF test results at a threshold of 10^-4^ or 10^-5^.^[5,6]^ Bone marrow and remaining peripheral blood samples were collected after clinical examination. The microvesicles were obtained through ultracentrifugation and labeled myeloma cell-derived microvesicles with the characteristic myeloma markers GPRC5D, BCMA, CD138, and CD319. The number of microvesicles was detected by flow-cytometry, as described in detail in our previous study^[4]^ (Supplementary Material S1, Supplementary Material S2). The differences in the numbers of microvesicles were compared between the L-TL and H-TL groups and the ROC curve analysis was performed on microvesicles to distinguish H-TL patients from L-TL patients.

**Figure 1:**
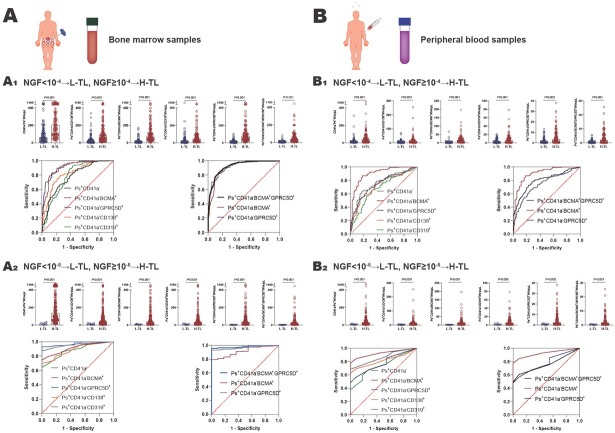
The value of microvesicles in bone marrow and peripheral blood in monitoring MM tumor load. A Bone marrow samples: A^1^ NGF = 10^-4^ as the threshold, the number of MM cell-derived microvesicles from bone marrow was significantly higher in H-TL group compared with in L-TL group, and the ROC curves were analyzed Ps^+^CD41a^−^, Ps^+^CD41a^−^CD138^+^, Ps^+^CD41a^−^BCMA^+^, Ps^+^CD41a^−^CD319^+^, Ps^+^CD41a^−^GPRC5D^+^, and Ps^+^CD41a^−^BCMA^+^GPRC5D^+^microvesicles from bone marrow to distinguish the H-TL patients from L-TL patients; A^2^ NGF = 10^-5^ as the threshold, the number of MM cell-derived microvesicles from bone marrow was significantly higher in H-TL group compared with in L-TL group, and the ROC curves were analyzed Ps^+^CD41a^−^, Ps^+^CD41a^−^CD138^+^, Ps^+^CD41a^−^BCMA^+^, Ps^+^CD41a^−^CD319^+^, Ps^+^CD41a^−^GPRC5D^+^, and Ps^+^CD41a^−^BCMA^+^GPRC5D^+^microvesicles from bone marrow to distinguish the H-TL patients from L-TL patients. B Peripheral blood samples: B_1_ NGF=10^-4^ as the threshold, the number of MM cell-derived microvesicles from peripheral blood was significantly higher in H-TL group compared with in L-TL group, and the ROC curves were analyzed Ps^+^CD41a^−^, Ps^+^CD41a^−^CD138^+^, Ps^+^CD41a^−^BCMA^+^, Ps^+^CD41a^−^CD319^+^, Ps^+^CD41a^−^ GPRC5D^+^, and Ps^+^CD41a^−^BCMA^+^GPRC5D^+^microvesicles from peripheral blood to distinguish the H-TL patients from L-TL patients; B_2_ NGF=10^-5^ as the threshold, the number of MM cell-derived microvesicles from peripheral blood was significantly higher in H-TL group compared with in L-TL group, and the ROC curves were analyzed Ps^+^CD41a^−^, Ps^+^CD41a^−^CD138^+^, Ps^+^CD41a^−^BCMA^+^, Ps^+^CD41a^−^CD319^+^, Ps^+^CD41a^−^GPRC5D^+^, and Ps^+^CD41a^−^BCMA^+^GPRC5D^+^microvesicles from peripheral blood to distinguish the H-TL patients from L-TL patients. NGF: next-generation flow; L-TL: low tumor load; H-TL: high tumor load; MVs: microvesicles; Ps: phosphatidylserine; AUC: area under the curve.

For bone marrow samples: when 10^-4^ was used as the threshold, the numbers of CD41a^-^Phosphatidylserine^+^(Ps^+^), Ps^+^CD41a^−^CD138^+^, Ps^+^CD41a^−^BCMA^+^, Ps^+^CD41a^−^CD319^+^, and Ps^+^CD41a^−^ GPRC5D^+^microvesicles were all higher in the H-TL group (*n* = 174) than the L-TL group (*n* = 137) (*P* < 0.001) (Figure 1A-A_1_ and Supplementary Table S2). Then, ROC curves were performed on bone marrow microvesicles labeled with Ps^+^CD41a^−^CD138^+^, Ps^+^CD41a^−^BCMA^+^, Ps^+^CD41a^−^CD319^+^, Ps^+^CD41a^−^GPRC5D^+^ to distinguish H-TL patients from L-TL patients. The AUC of these microvesicles were 0.8222, 0.9054, 0.7812, 0.9159 (*P* < 0.001), with sensitivities of 78.74%, 83.24%, 93.10%, 89.66%, and specificities of 75.18%, 86.13%, 56.93%, 78.83%, respectively (Figure 1A-A_1_ and Table 1). It suggested that BCMA and GPRC5D-positive microvesicles can reflect MM tumor load well in bone marrow (AUC > 0.9).

**Table 1:** The AUC values of ROC curves

				Optimal		
Group		AUC (95%CI)	*P* value	Cut off points, μL	Sensitivity, % (95%CI)	Specificity, % (95%CI)
< 10^-4^ *vs*. ≥ 10^-4^ (Bone marrow)	CD41a-Ps+	0.7733 (0.7210-0.8255)	< 0.001	134.3	66.67 (59.37–73.24)	75.18 (67.33–81.66)
	Ps+CD41a-CD138+	0.8222 (0.7757-0.8688)	< 0.001	35.55	78.74 (72.07–84.16)	75.18 (67.33–81.66)
	Ps+CD41a-BCMA+	0.9054 (0.8712-0.9396)	< 0.001	30.09	83.24 (76.96–88.07)	86.13 (79.35–90.94)
	Ps+CD41a-CD319+	0.7812 (0.7277-0.8346)	< 0.001	13.90	93.10 (88.33–96.01)	56.93 (48.57–64.93)
	Ps+CD41a-GPRC5D+	0.9159 (0.8852-0.9467)	< 0.001	21.18	89.66 (84.24–93.36)	78.83 (71.25–84.84)
	Ps+CD41a-BCMA+GPRC5D+	0.9248 (0.8960-0.9536)	< 0.001	17.16	80.46 (73.79–85.67)	90.51 (84.44–94.37)
< 10^-5^ *vs*. ≥ 10^-5^ (Bone marrow)	CD41a-Ps+	0.9691 (0.9448-0.9933)	< 0.001	30.83	89.67 (85.71–92.62)	63.64 (35.38–84.83)
	Ps+CD41a-CD138+	0.8855 (0.8249-0.9460)	< 0.001	20.85	73.33 (68.06–78.02)	81.82 (52.30–96.77)
	Ps+CD41a-BCMA+	0.9133 (0.8643-0.9624)	< 0.001	10.50	79.00 (74.04–82.23)	90.90 (60.20–98.30)
	Ps+CD41a-CD319+	0.8818 (0.8179-0.9457)	< 0.001	16.81	68.00 (62.52–73.02)	72.73 (43.44–90.25)
	Ps+CD41a-GPRC5D+	0.9715 (0.9507-0.9923)	< 0.001	1.840	93.00 (89.54–95.38)	90.91 (62.26–99.53)
	Ps+CD41a-BCMA+GPRC5D+	0.9800 (0.9646-0.9954)	< 0.001	1.005	95.67 (92.73–97.45)	90.91 (62.26–99.53)
< 10^-4^ *vs*. ≥ 10^-4^ (Peripheral blood)	CD41a-Ps+	0.7564 (0.6954-0.8175)	< 0.001	25.78	64.89 (56.39–72.53)	81.08 (72.80–87.28)
	Ps+CD41a-CD138+	0.7242 (0.6605-0.7880)	<0.001	10.23	52.67 (44.17–61.02)	84.68 (76.84–90.21)
	Ps+CD41a-BCMA+	0.9015 (0.8620-0.9409)	< 0.001	4.570	87.20 (80.20–91.74)	81.98 (73.80–88.02)
	Ps+CD41a-CD319+	0.6855 (0.6185-0.7525)	< 0.001	3.640	64.12 (55.61–71.83)	69.37 (60.27–77.18)
	Ps+CD41a-GPRC5D+	0.7470 (0.6857-0.8082)	< 0.001	2.150	64.89 (56.39–72.53)	74.77 (65.96–81.93)
	Ps+CD41a-BCMA+GPRC5D+	0.7961 (0.7400-0.8521)	< 0.001	0.985	72.52 (64.32–79.77)	77.48 (68.86–84.25)
< 10^-5^ *vs*. ≥ 10^-5^ (Peripheral blood)	CD41a-Ps+	0.8617 (0.7889-0.9344)	< 0.001	12.66	70.13 (63.94–75.66)	72.73 (43.44–90.25)
	Ps+CD41a-CD138+	0.8245 (0.7583-0.8907)	< 0.001	3.430	65.37 (59.03–71.21)	81.82 (62.26–99.53)
	Ps+CD41a-BCMA+	0.9282 (0.8876-0.9687)	< 0.001	3.415	65.37 (59.03–71.21)	84.85 (74.12–99.00)
	Ps+CD41a-CD319+	0.7320 (0.6295-0.8345)	0.009	5.005	43.72 (37.48–50.17)	63.64 (43.44–84.83)
	Ps+CD41a-GPRC5D+	0.7719 (0.6772-0.8667)	0.002	1.560	55.41 (48.96–61.68)	81.82 (52.30–96.77)
	Ps+CD41a-BCMA+GPRC5D+	0.7757 (0.6901-0.8613)	0.002	0.725	58.87 (52.43–65.02)	99.91 (74.12–99.53)

AUC: area under the curve; Ps: phosphatidylserine.

In our previous study, we found the double-positive microvesicles (such as Ps^+^CD41a^−^ CD138^+^BCMA^+^microvesicles) were less valuable in monitoring MM tumor load than single-positive microvesicles (such as Ps^+^CD41a^−^CD138^+^ and Ps^+^CD41a^−^ BCMA^+^). This is mainly because the number of double-positive microvesicles extremely reduced.^[4]^ However, the expression of GPRC5D and BCMA in myeloma are mutually exclusive.^[5,7]^ Thus, we explored whether Ps^+^CD41a^−^ BCMA^+^GPRC5D^+^microvesicles could be a better marker. The AUC of Ps^+^CD41a^−^BCMA^+^GPRC5D^+^microvesicles was 0.9248, with sensitivity of 80.46% and specificity of 90.51% (Figure 1A-A_1_, Table 1). It suggested that the number of Ps^+^CD41a^−^BCMA^+^GPRC5D^+^microvesicles in bone marrow is a better marker of MM tumor load (AUC value maximum). Mechanistically, we inferred that during vesicle formation, BCMA-positive microvesicles and GPRC5D-positive microvesicles may be more likely to fuse with each other, although the expression of BCMA and GPRC5D in myeloma cells are mutually exclusive. Therefore, the mutually exclusive expression on cells may lead to dual-positive microvesicles representing a broader tumor subpopulation. Of course, this hypothesis requires further research to confirm in the future.

When 10^-5^ was used as the threshold, the numbers of Ps^+^CD41a^-^, Ps^+^CD41a^−^CD138^+^, Ps^+^CD41a^−^BCMA^+^, Ps^+^CD41a^−^CD319^+^, Ps^+^CD41a^−^GPRC5D^+^ and Ps^+^CD41a^−^ BCMA^+^GPRC5D^+^microvesicles were all higher in H-TL group (*n* = 300) than L-TL group (*n* = 11)(*P* < 0.001) (Figure 1A-A_2_, Supplementary Table S2). And the AUC of these microvesicles were 0.8855, 0.9133, 0.8818, 0.9715, 0.9800 (*P* < 0.001), with sensitivities of 73.33%, 79.00%, 68.00%, 93.00%, 95.67%, and specificities of 81.82%, 90.90%, 72.73%, 90.91%, 90.91%, respectively (Figure 1A-A_2_, Table 1).

For peripheral blood samples: when NGF = 10^-4^ was used as the threshold, the numbers of CD41a^−^Ps^+^, Ps^+^CD41a^−^CD138^+^, Ps^+^CD41a^−^BCMA^+^, Ps^+^CD41a^−^ CD319^+^, Ps^+^CD41a^−^GPRC5D^+^ and Ps^+^CD41a^−^ BCMA^+^GPRC5D^+^microvesicles were all higher in H-TL group (*n* = 111) than L-TL group (*n* = 131) (*P* < 0.001) (Figure 1B-B_1_, Supplementary Table S2). The AUC of these microvesicles were 0.7242, 0.9015, 0.6855, 0.7470, 0.7961 (*P* < 0.001), with sensitivities of 52.67%, 87.20%, 64.12%, 64.89%, 75.52%, and specificities of 84.68%, 81.98%, 69.37%, 74.77%, 77.48%, respectively (Figure 1B-B_1_, Table 1). When NGF = 10^-5^ was used as the threshold. The numbers of these microvesicles were also significantly higher in H-TL group (*n* = 111) than in L-TL group (*n* = 131) (*P* < 0.01) (Figure 1B-B_1_, Supplementary Table S2). And the AUC of these microvesicles were 0.8245, 0.9282, 0.7320, 0.7719, 0.7757 (*P* < 0.01), with sensitivities of 65.37%, 65.37%, 43.72%, 55.41%, 58.87%, and specificities of 81.82%, 84.85%, 63.64%, 81.82%, 99.91%, respectively (Figure 1B-B_2_, Table 1). We noticed that the lower AUC score for peripheral blood MVs compared to that for bone marrow, which suggested that peripheral blood may be less reliable. We believed it may be related to the dilution effect of peripheral blood relative to bone marrow, the half-life of microvesicles, and the depletion of microvesicles in peripheral blood samples during storage.

In conclusion, the number of microvesicles in both bone marrow samples and peripheral blood samples can reflect MM tumor load and serve as a useful complement to existing tumor load monitoring methods to compensate for false negatives. Specifically, when using bone marrow samples, the selection of Ps^+^CD41a^−^BCMA^+^, Ps^+^CD41a^−^GPRC5D^+^, and Ps^+^CD41a^−^BCMA^+^GPRC5D^+^ microvesicles were all effective in reflecting tumor load, with AUC above 0.9, and Ps^+^CD41a^−^BCMA^+^GPRC5D^+^microvesicles were the most effective, with AUC maximum; when using peripheral blood samples, the selection of Ps^+^CD41a^−^BCMA^+^microvesicles can reflect the tumor load better, with AUC above 0.9. It appears that BCMA can be used as an effective member in bone marrow and peripheral blood samples, but GPRC5D combined BCMA can be more effective than BCMA alone in bone marrow. The underlying mechanisms still need further study. However, the current results are only based on the single center data. It is crucial to establish a standard system. We hope to conduct further research using data from multiple centers, and to follow up over a longer period. Furthermore, clear standards need to be established for sample collection, storage, preparation, and flow cytometry monitoring strategies, all these standards should be validated across multiple laboratories. These will facilitate the clinical application of this technology. To create a standard system for microvesicle detection can more usefully promote the monitoring of MM tumor load in the future.

## Supplementary Material

Supplementary Material Details
