# Supplementary material for: Microvesicles derived from bone marrow and peripheral blood can reflect tumor load in multiple myeloma
Source: J Transl Int Med. 2025 Dec 29;14(1):162–5. doi: 10.1515/jtim-2025-0096 (PMC12916271; doi:10.1515/jtim-2025-0096)
Supplement: Supplementary file 1 — Supplementary Material Details [file jtim-2025-0096_sm.pdf]

Supplementary Materials

Microvesicles derived from bone marrow and peripheral blood can reflect tumor load in multiple myeloma

Nanhao Meng[1,2], Zhaoyun Liu[1,2], Yan Shi[1,2], Chun Yang[1,2], Hao Wang[1,2], Yanran Luo[1,2], Hui Liu[1,2], Kai Ding[1,2], Fengjuan Jiang[1,2], Fengping Peng[1,2], Rong Fu[1,2]*

[1]Department of Hematology, Tianjin Medical University General Hospital, Tianjin 300052, China

[2]Tianjin Key Laboratory of Bone Marrow Failure and Malignant Hemopoietic Clone Control, Tianjin Institute of Hematology, State Key Laboratory of Experimental Hematology, Tianjin 300020, China

Address for Correspondence: Rong Fu, Department of Hematology, Tianjin Medical University General Hospital, 154 Anshan Street, Heping District, Tianjin 300052, China. E-mail: Furong8369@tmu.edu.cn

Supplementary material 1-Materials and Methods

Antibodies and reagents

The antibodies and reagents used here included: Annexin V-fluorescein isothiocyanate (BD Horizon, cat no. 51-65874X), anti-CD138-APC (clone: 44F9, lot no. 5190701751), anti-BCMA-Percp/Cyanine5.5 (clone: 19F2, lot no. 13288964), anti-CD319-PE (clone: REA150; lot no.5190924560), anti-GPRC5D-PE (clone: 571961R; Catalog: FAB6300RP), and anti-CD41a-PE-Vio770 (clone: HIP8; lot no. 5190924444). The latex beads with a diameter of 0.22 μm, 0.45 μm, 0.88 μm, and 1.35 μm (cat no. NFPPS-52-4K) were all purchased from Spherotech, Germany.

Study design and patient selection criteria

Samples were collected from 311 MM patients (>18 years of age). The gender (male/female) of MM patients was 175/136 *(Supplementary material 3-Supplementary Table1)*. Patients with CR are defined via two consecutive assessments' demonstrating negative serum and urine immunofixation electrophoresis, with <5% bone marrow plasma cells. Certain patients' results, based only on serum free light chain, were accepted upon their return to normal free light chain ratios.

Isolation of microparticles from the bone marrow and peripheral blood

Up to 4 mL of Ethylenediaminetetraacetic acid (EDTA) bone marrow and peripheral blood was centrifuged at 1500×*g* for 30 min at room temperature (RT). The precipitate was discarded to obtain platelet-poor plasma, followed by centrifugation at 13,000×*g* for 2 min at RT to obtain platelet-free plasma (PFP) from the supernatant. The PFP was divided into 200μL aliquots, which were subjected to microparticle isolation by ultracentrifugation at 18890×*g* at 4 °C for 30 min. Then the supernatant was removed, and the MP pellet was immunolabeled for flow cytometry. It is strongly recommended

that MP centrifugation and flow cytometry be performed within 4 hours of obtaining bone marrow or peripheral blood samples. If immediate analysis is not possible, the obtained MPs pellet should be stored at -80°C until analysis, and the analysis should be completed within 1 month. All the frozen samples were thawed on ice before immunolabelling. Technical triplicates were performed for each patient's microvesicles count to reduce error.

Flow cytometry

All the phenotyping and counting of microvesicles were performed using a Cyto-Flex flow cytometer (Beckman Coulter, USA). An MP gate was set on the 2D scatter plot of FSC-A *vs*. Violet SSC-A. Latex beads of 0.22 μm, 0.45 μm, 0.88 μm, and 1.35 μm in diameter respectively, were used to define the range of MP gate. Compared to latex beads, microvesicles have lower refractive indices and their diameter is too small to be detected using conventional methods, therefore, we adjusted the side scatter (SSC) to 405nm (instead of 488nm) laser light to improve sensitivity of MP detection and avoid background noise during acquisition. This predefined MP gate was applied to all samples. $CD41a^{-}$, Annexin $V^{+/-}$, $CD138^{+/-}$, $BCMA^{+/-}$, $CD319^{+/-}$, and $GPRC5D^{+/-}$ were applied to MP populations. All fluorescence positivity gates were defined based on the background fluorescence in isotype and negative controls *(Supplementary material 2-Supplementary Figure)*. Before each sample was run, the flow cytometer was washed with deionized water until the number of particles was <100/s, thus reducing interference from possible impurities. Samples were run at low flow rate (about 10-30μL/min) to reduce the occurrence of coincidence. If the sampling rate was high (particle count was >10000/s) and the dropout rate is also high, the "acquisition rate setting" was adjusted to "high". Compensation matrices applying and data analyzing were used CytExpert Analysis Software (Beckman Coulter, USA).

Surface protein phenotyping of microparticles

CD41a, Annexin V, CD138, BCMA, CD319, and GPRC5D were used to immunolabel microvesicles isolated from bone marrow and peripheral blood of MM patients in parallel with relevant isotype and negative controls. Firstly, the isolated MP pellet was immunolabelled with 5μL of Annexin V-FITC, and placed in the dark for 5 min at RT. Secondly, to exclude the effect of platelet-derived microvesicles, the isolated MP pellet was labelled with 5μL of anti-CD41a-Vio770 for 30 min in the dark at RT. At the same time, using 5μL of anti-CD138-APC, 5μL of anti-BCMA- Percp/Cyanine5.5, 5μL of anti-CD319-PE, and 5μL of anti-GPRC5D-PE labelled MP pellet for 30 min in the dark at RT. The isotype controls were multiply labelled for 30 min in the dark at RT, using 5 μL of anti-IgG1-PE-Vio770, 5 μL of anti-IgG1-APC, 5 μL of anti-IgG1-Percp/Cyanine5.5, and 5μL of anti-IgG1-PE. While the negative controls were only labelled by 5μL of Annexin V-FITC. Thirdly, the 10×Bunding buffer was diluted to 1× at 1:9, then 200μL of diluted buffer was add into per 100μL labeled sample. This step is indispensable because the Calcium (Ca^{2+}) dependence of Annexin V.

Statistical analysis

Statistical analyses were conducted using IBM SPSS Statistical version 20.0 for Windows (IBM SPSS Software, RRID: SCR_002865, Chicago, IL, USA, www.ibm.com). Statistical graphs were drawn using GraphPad Prism version 8.0.2 for Windows (GraphPad Software, RRID: SCR_002798, San Diego, California, USA, www.graphpad.com). The Shapiro−Wilk test was used to assess the distribution normality of data for each group. The Mann-Whitney U test was used for nonparametric data, and the data were presented as the median (Q1, Q3), and Mann-Whitney constant

U and p values were stated. All the data were expressed as numerical variables. The results were considered statistically significant at predictive values of $P<0.05$.

Supplementary material 2-Supplementary Figure

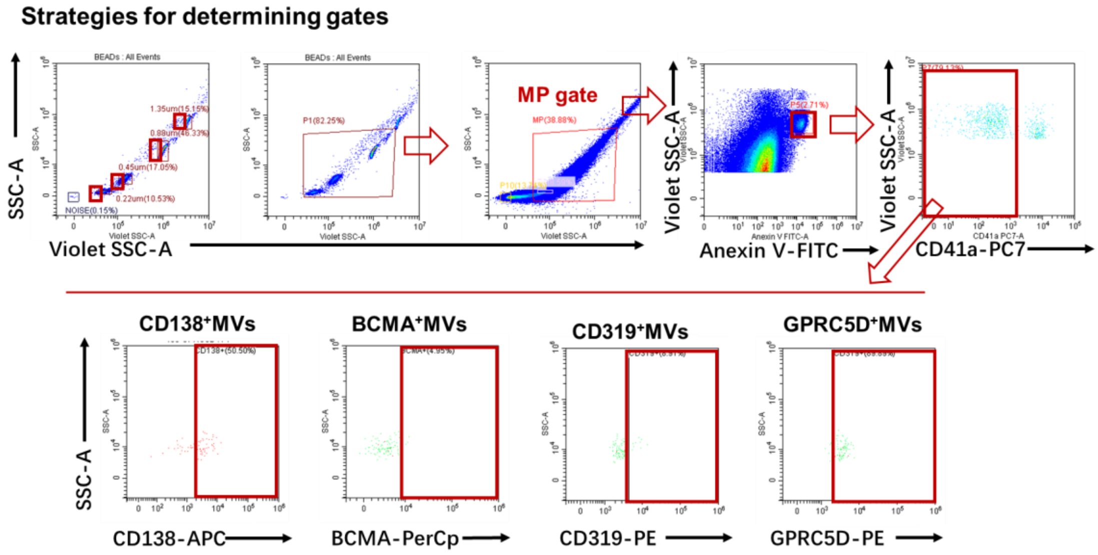

Supplementary Figure S1

The strategy for analyzing microvesicles via flow cytometry. Isolating microvesicles by differential ultracentrifugation from bone marrow. Using the standardized diameter latex beads (0.22 µm, 0.45 µm, 0.88 µm, and 1.35 µm) to identify the "MVs Gate", Annexin V-FITC was used to distinguish Ps^+ microvesicles in "MVs Gate", and CD41a-PEYC7 was used as a marker to remove the platelet-derived microvesicles and obtain $CD41a^-Ps^+$ microvesicles. Then $CD41a^-Ps^+$ microvesicles were set as the parent groups, and $CD138^+$, $BCMA^+$, $CD319^+$, $GPRC5D^+$ microvesicles were analyzed.

Supplementary material 3-Supplementary Tables

Table S1 Characteristics of the patients

Group	N (%)
Total no. of patients	311 (100)
Gender (M/F)	175/136 (56/44)
Age, median (Q1-Q3)	66 (42-81)
Type of MM	IgG 202(65.1), IgA 95(30.5), IgM 5(1.6), IgE 2(0.6), Light chain 5(1.6), nonsecretory 2(0.6)

Table S2 The numbers of microvesicles in different tumor load group

Bone marrow		NGF		*P* value
		$<10^{-4}$	$\geq10^{-4}$	
N		137	173	
Ps+	/	61.02 (29.89, 137.77)	204.7 (96.82, 435.26)	<0.001
	CD138+	20.68 (9.92, 35.69)	83.44 (38.48, 166.14)	<0.001
	BCMA+	10.46 (4.25, 21.98)	79.96 (41.86, 159.27)	<0.001
	CD319+	12.46 (4.56, 35.77)	45.34 (22.77, 97.64)	<0.001
	GPRC5D+	12.00 (4.07, 20.58)	78.85 (37.49, 141.06)	<0.001
	BCMA+GPRC5D+	4.32 (1.04, 10.24)	39.12 (20.12, 82.43)	<0.001
Bone marrow		NGF		*P* value
		$<10^{-5}$	$\geq10^{-5}$	
N		11	300	
Ps+	/	12.36 (2.43, 18.76)	134.3 (59.39, 319.23)	<0.001
	CD138+	8.1 (1.01, 16.00)	42.88 (19.66, 101.79)	<0.001
	BCMA+	3.75 (0.46, 8.24)	39.99 (14.38, 98.96)	<0.001
	CD319+	5.07 (1.02, 10.70)	30.55 (13.00, 75.84)	<0.001
	GPRC5D+	0.93 (0.08, 1.27)	35.10 (15.03, 89.96)	<0.001
	BCMA+GPRC5D+	0.15 (0.02, 0.95)	18.93 (6.39, 48.20)	<0.001
Peripheral blood		NGF		*P* value
		$<10^{-4}$	$\geq10^{-4}$	
N		111	131	
Ps+	/	12.71 (6.43, 22.98)	41.45 (15.90, 78.26)	<0.001

	CD138+	2.60 (1.20, 7.75)	10.98 (4.27, 20.90)	<0.001
	BCMA+	1.48 (0.51, 3.91)	13.32 (6.71, 23.86)	<0.001
	CD319+	1.44 (0.47, 4.98)	6.47 (1.66, 11.32)	<0.001
	GPRC5D+	0.89 (0.31, 2.31)	3.61 (1.20, 8.96)	<0.001
	BCMA+GPRC5D+	0.24 (0.08, 0.98)	2.06 (0.93, 5.67)	<0.001
Peripheral blood		NGF		*P* value
		$<10^{-5}$	$\geq 10^{-5}$	
N		11	231	
Ps+	/	6.54 (1.46, 10.26)	22.94 (10.70, 54.57)	<0.001
	CD138+	1.26 (0.50, 2.00)	7.18 (2.10, 15.28)	<0.001
	BCMA+	0.27 (0.05, 0.60)	6.04 (1.91, 19.54)	<0.001
	CD319+	1.20 (0.24, 2.21)	3.71 (0.81, 10.07)	0.008
	GPRC5D+	0.79 (0.04, 1.00)	2.09 (0.57, 5.65)	0.002
	BCMA+GPRC5D+	0.20 (0.02, 0.35)	1.02 (0.20, 3.21)	0.001

Data are expressed as median (Q1, Q3)

NGF: next-generation flow; Ps: phosphatidylserine
